# Diphtheria

**Published:** 1881-01

**Authors:** Francis Delafield

**Affiliations:** Adjunct Professor of Pathology and Practical Medicine in the College of Physicians and Surgeons, New York


ATLANTA

Medical and Surgical Journal.

VOL. XVIII.] JANUARY—1881. [No. 10.

### Original Communications.

### DIPHTHERIA.—A LECTURE.

BY FRANCIS DELAFIELD, M.D.
Adjunct Professor of Pathology and Practical Medicine in the College of Physicians and Surgeons, New York.

[Reported for Atlanta Medical and Surgical Journal.]

*Gentlemen.*—We will continue the subject of diphtheria. I told you that the lesion is a croupous inflammation of certain of the mucous membranes of the body. The mucous membranes which are most frequently affected are those covering the tonsils, the pharynx, the soft palate, the posterior nares, the larynx, and the trachea, less frequently we have the mucous membrane which lines the mouth, mucous membrane covering the gums, the œsophagus, the stomach, and the vagina affected in the same way. The extent of the lesion, I told you, varies a good deal in the different cases. In some cases there is simple congestion of the mucous membrane, and there are small patches of false membrane scattered here and there over this congested and swollen membrane. In other cases there is also the general congestion of the mucous membrane, but to the naked eye there may not appear any false membrane at all, not because the false membrane is not present, but because it is so thin and so scanty that it is not appreciated; then, in still other cases, we have a continuous, thick layer of false membrane covering a considerable surface. In still other cases with this production of false membrane, the infiltration of the soft parts beneath is very marked, and in still other cases the inflammatory process is accompanied by death of circumscribed portions of tissue, and these circumscribed portions of tissue slough away if the patient live long enough. The glands of the neck, both the lymphatic and the salivary glands, may be swollen or not. In a certain number of cases the swelling goes on to actual suppurative inflammation, and abcesses are formed in the neck which may be discharged. There may be a complicating bronchitis, or a complicating pneumonia, and the bronchitis may be a catarrhal bronchitis or a croupous bronchitis; the pneumonia may be either lobar pneumonia or lobular pneumonia. In a certain number of cases the inflammatory process extends to the œsophagus and the stomach, and they too are coated with false membrane. The kidneys, in a very considerable number of cases, are the seat of parenchymatous nephritis.

As to the causation of the disease, I told you that our knowledge is still uncertain. We know that it occurs in epidemics, and we know that in any given place where it has occurred as an epidemic it may remain as an epidemic disease. We know that it occurs much more frequently among children than it does among adults, and we know that it occurs especially, in New York at least, in children of about the age of five years. There also seems to be good reason for believing that the disease is directly contagious, that it can be transmitted from one person to another, although we are still ignorant of the exact way in which this contagion is affected. We also have to admit the existence of idiopathic cases—of cases which occur without any cause which we are able to find out.

As to the nature of the disease, I told you that there are two principal theories prevailing. One is, that the disease is a general disease with a characteritic local lesion, a disease like typhoid fever or typhus fever. Another opinion is, that the disease at first is a local lesion, a simple affection of the throat, and that the system is poisoned by matters absorbed from the inflamed throat.

As regards the symptoms of the disease, I told you that we have in the first place those distinguishing the malignant cases, and of these malignant cases there are two varieties. In the first place, those cases which occur with an acute invasion. There are rigors rapidly followed by a febrile movement. There may be one or more convulsions marking the invasion of the disease. In a few hours either one of the tonsils, or both tonsils, or the whole pharynx becomes congested and swollen. By the end of twelve or twenty-four hours the false membrane is produced, either on one of the tonsils, or on some part of the pharynx which we are able to recognize. The febrile movement continues, the false membrane continues to become more and more extensive, and to cover more and more of the throat. The functions of the kidneys are interferred with, the urine becomes albuminous, often scanty, sometimes almost suppressed. There may be swelling of the lymphatic and salivary glands of the neck. The patient developes cerebral symptoms, becomes delirious, becomes drowsy, becomes comatose, and dies in this condition, and dies usually at the end of three or five days. In this case the progress of the disease is nearly continuous. The patient gets worse from hour to hour, and from day to day. There is no let up whatever in the symptoms

In the second set of malignant cases the invasion of the disease is not acute, it is not marked, it is insidious, and a little slow. The child at first seems to be a little out of sorts. It loses its appetite, it does not wish to play, it does not care to do anything except to sit still, or to lie on the bed, or to lie on the sofa. It becomes fretful and peevish, but there is no febrile movement. The child does not complain of its throat. If you look at the throat you do not see anything at all, or you may simply see a little congestion. Then the next thing that you notice is, that the neck becomes somewhat swollen, the swelling being due either to a swelling of the salivary glands or to the lymphatic glands; and now if you look at the throat the congestion may be still more marked, but still the child will very often not complain of its throat at all. It will complain of the swelling in the neck, but it will tell you that it has no pain from the swelling, that its throat does not pain it at all. The pulse now becomes more frequent and feeble; the skin is pale and cool. Now there begins to be discharged a dirty brownish fluid, which runs from the mouth and from the nose. It runs from the mouth and nose especially when the child is lying down, and when it is asleep. If you lift the child's head and look at the pillow on which it has been lying you will see that it is discolored by this thin, dirty, brown fluid, which has run away either from the mouth or the nose, or from both. In this condition, without any more symptoms than these, the child may continue for a few days, for three or four days, or even for a couple of weeks, and during all this time there will be no febrile movement, there will be no membrane, which you can see, in the throat, there will be nothing but the general depression of the child's condition, the feeble heart action and the escape of this dirty, brownish fluid from the mouth and nose. There may, in addition to this, be albumen in the urine. Then, at the end of this time, at the end of the three or four days, or at the end of the two weeks, the child dies, and it usually dies suddenly. You may see it in just the condition which I have described, looking like a child which is quite ill and yet not looking as if there was anything to cause sudden death, and within half an hour or an hour after you see it the child will suddenly die. If it is lying down its breathing will become a little quicker, a little more rapid, then slower, then it will stop and the child will be dead. Occasionally these children will die so suddenly that even the people who are taking care of them are not aware of it until sometime afterwards. This is especially the case during the night If the child is sleeping in the bed along side of its mother, or along side of its nurse, it will be quiet, it will apparently go to sleep, the mother or the nurse will go to sleep; in the course of the night they will wake up, they will look at the child, they will see that it does not move, and they will find that the child is dead, is cold, is rigid, and evidently has been dead sometime before they found it out. Death occurs in these children in an unexpected way. In these cases, although there is no false membrane which you can see during the life of the child, yet the characteristic lesion is present. There is a croupous inflammation, sometimes confined to the larynx, sometimes involving the pharynx, the tonsils, and the larynx, but the amount of this membrane produced is very small, it is so small that, although it is there, it escapes observation. These are the two varieties of the malignant cases.

The more ordinary form of the disease—the form which we see, and have seen for some years, most frequently in New York—occurs more in this way: The first symptom complained of is the sore throat. The child or the adult complains of a feeling of soreness in the throat. If you look at the throat you will find that there is a general congestion, and you will find either on one of the tonsils or on some part of the pharynx a small patch of false membrane. At the same time there may or may not be a febrile movement. This varies in the different cases. In a certain number of cases the febrile movement will come on a little before, or with the feeling of soreness in the throat. In other cases the sore throat will be the first symptom complained of, and there will be no febrile movement developed until after several days. Only this fall one of the house staff at Bellevue Hospital, who died of the disease, complained at first of a feeling of soreness in the throat. He was on my division in the hospital, and one day, after he had been going the rounds, he asked me to look at his throat, and there was a large white patch on one of the tonsils. I told him to go home and go to bed, which he did. But the throat continued to get worse; but there was no febrile movement until the third or fourth day; I have forgotten which, and he died on the eighth day. There is, then, a great variety as to the existence or non-existence of fever at the beginning of the disease. The false membrane gradually extends. It extends to more and more of the throat, until finally, in many cases, all of the throat that you will be able to see is covered by a thick layer of false membrane. The false membrane is at first white, afterwards it becomes of a grey or of a dirty brown color. With this there may or may not be a swelling of the glands of the neck. This is sometime present; sometimes absent; there may or may not be disturbance of the stomach; some of the patients vomit; some of them simply complain of a little nausea; some of them have neither vomiting nor nausea. There is always a certain amount of prostration; the patients feel sick; they feel weak; they are disposed to go to bed and to keep quiet, although to this there are exceptions in the first few days of the disease. There are persons who will be walking about and attending to their business during the first three or four days after the development of, or with the commencement of the development of the false membrane in the throat. If we examine the urine we find that in a certain number of cases it is albuminous. This is the rule. But there are exceptions to this rule. We also find in a certain number of cases that the urine is diminished in amount; that it is scanty. The disease in these cases seems to progress pretty steadly for about a week; for about six or seven days. During all this time you will find the false membrane constantly extending and involving more and more of the surface. You will find the patient's general condition becoming worse and worse; you will find the febrile movement (if it were present at the beginning) continue, or if it were not present at the beginning, developing about the third, fourth or fifth day. Then, at the end of this time, at the end of the first week—sometimes a day before, sometimes a day after—comes the danger of an extension of the inflammation to the larynx. It is about this time, from the fifth to the eighth day, that we have to expect that the inflammatory process may extend to the larynx, and that the larynx too will be covered by the false membrane. If this is the case the patient's symptoms at once become a good deal worse. There is difficulty in breathing, and in addition to the difficulty in breathing, it becomes evident that the patient is much sicker; the prostration is more marked; there may be delirium. At this time there may be drowsiness, alternating with delirium. In those cases in which the inflammatory process does not extend to the larynx; the end of the fiist week is the time at which the inflammatory process usually seems to have run its course, and now we begin to have a detachment of the false membrane. The false membrane begins to come away. This detachment of the false membrane is often accompanied with an increased function of the mucous glands of the parts, so that there is a very profuse flow of mucus, and the patient will be constantly coughing out quantities of mucus, and with this mucus will come away fragments of the false membrane. After this the patient will usually go on and get better. The throat will gradually clear off, and, by the end of two weeks, sometimes a little longer, convalescence will fairly set in. In those cases in which the lesion extends to the larynx, however, the condition of affairs is much worse. There is, as I said, a well marked difficulty in breathing; there is a well marked prostration on the part of the patient; there may be general symptoms. This condition, in a very large number of cases, continues and becomes fatal. The breathing becomes more and more difficult; the cerebral symptoms become more and more marked, and the patient dies at the end of a few hours or a few days, after the development of the laryngeal symptoms. In other cases, however, the progress of the disease is more favorable; we have the dyspnœa; we have the prostration; this continues for one, or two, or three, or four days, and yet the patient does succeed well in breathing; then the false membrane begins to loosen; the patient begins to cough it up; the dyspnœa gradually diminishes; the cerebral symptoms disappear; the general condition of the patient improves, and again, by the end of two weeks or longer, convalescence will fairly set in.

In these cases, which represent the ordinary run of cases as we see them in New York, the prognosis depends very much indeed upon the presence or absence of the laryngeal complication. If the disease does not occur in too young a child, the cases in which the disease does not involve the larynx recover to a considerrble extent; they don't all recover, but a very considerable number of them recover. To the extent in which the larynx becomes involved the prognosis is bad; very much the larger number die; still, even in these cases, as I say, the prognosis is not hopeless. It is always possible for the membrane to become detached and to be coughed up before the difficulty of breathing has become so great as to cause death. Again, we meet with cases in which the lesion is confined to the larynx. The pharynx and the tonsils are not involved; the patient does not complain of sore throat; in these cases the symptoms are just like the symptoms of croupous laryngitis; you have the same symptoms that you have in croupous laryngitis; you have the febrile movement more or less developed; you have the laryngeal local symptoms; the cough; the stridulous breathing; the stridulous voice; the loss of voice; the dyspnœa constantly becoming worse and worse; the development of carbonic acid poisoning; the cerebral symptoms, and the death of the patient.

In other cases, again, the disease runs a very mild course indeed; the patient is not at any time very sick; the throat lesions are not at any time very marked; there is a patch of false membrane on one of the tonsils, and this patch does not spread; it remains confined to the tonsil; or, there may be a patch at some part of the mucous membrane of the pharynx, and this patch again does not spread, but remains confined to a moderate area; and the constitutional symptoms in these cases are frequently slight; there may be no febrile movement, or a very slight febrile movement; and, after lasting for five, six, or seven days, the patch or false membrane becomes detached. It is coughed away; it is expectorated; the throat returns to the natural condition, and the patient gets well, without at any time having been very sick, sometimes without at any time being confined to the bed.

In still other cases the disease begins in the same slight way; there is only a small patch of false membrane at some part of the throat; there are only very slight constitutional symptoms, and in this condition the patient may continue for several days, sometimes for a week, and you will think that the case is going to be one of the mild ceses; one of the cases in which the patient is not very sick at any time. But after continuing in this condition for several days, or for a week, the patient will suddenly become worse. This change in the condition of the patient will come on very rapidly indeed, and the principal change will be in the constitutional symptoms. There will suddenly be very marked prostration, either with or without the development of the febrile movement; the throat will also become more inflamed; the false membrane will extend, and is especially apt in these cases to extend to the larynx, and these patients will develop laryngeal symptoms; will become very ill inded, and sometimes, quite frequently, the cases will end fatally, although they began in the mild way.

In all the different varieties of the disease there is always danger of the sudden death of the patient from syncope; from failure of the heart's action; this danger exists as well in the mild cases as in the severe cases. In the severe cases the danger is especially marked just at the time when convalescence is beginning; just at the time when the patients are apparently getting better; then it is always possible, no matter how well the patients seem to be doing, for them to die suddenly from syncope. As I say, this danger exists even in mild cases, even in patients who are so little sick that they are not confined to bed.

Let us consider the individual symptoms of the disease. In the first place, with reference to the membrane, this appears first on one of the tonsils, or on some part of the pharynx, or in the posterior nares, or in the larynx, and after beginning in one or other of these situations, it spreads in the largest number of cases; it extends and involves the adjacent parts. In a certain number of cases, especially malignant cases, there is an escape of the dirty, brownish fluid from the mouth and from the neck; and in a certain number of cases there is a bleeding from the mouth, from the nose, or from the gums, the glands of the neck, either the lymphatic glands or the salivary glands are swollen in some cases; not swollen in others. In the cases in which they are swollen, the degree of swelling varies very much, and, in a certain number of cases, the swelling is succeeded by a suppurative inflammation.

Rigors mark the invasion of the disease in a moderate number of cases, not in a very large number; the febrile movement may be absent throughout the whole course of the disease; it may be present at the commencement of the disease, and continue nearly up to the time of the patient's death; it may be present at first and then subside, or it may be absent at first and then appear several days after the development of the throat trouble. These are the different varieties of the febrile movement; absent altogether throughout the whole course of the disease; present at the first, and then subside, although the other symptoms continue; or absent at first, and not appear until several days after the development of the throat lesion.

Symptoms referable to the larynx are only present after the larynx is involved in the inflammatory process. When this is the case we have the regular symptoms of croupous laryngitis. We have the laryngeal voice; the stridulous voice; the stridulous cough; the loss of voice; the stridulous breathing, and the dyspnœa due to the obstruction of the larynx. In the larger number of cases, the obstruction is so great as to prove fatal; in a smaller number of cases the obstruction is more moderate, and the patients are able to continue to live until the inflammatory process has run its course and subsided.

Vomiting is present sometimes early in the disease; it is not developed until late in the disease, and in some cases there is no vomiting at any time. But in the majority of cases, if there is no vomiting, there is at least some nausea; it is easy always to make these patients vomit; in a few cases the bowels are disturbed; there is a diarrhœa; this seems to be rather an accidental symptom, not to belong properly to the disease.

The pulse usually corresponds to the febrile movement. In those cases in which there is a well marked febrile movement at the commencement of the disease, the pulse is moderately rapid and full. In those cases in which there is no febrile movement, the pulse is more feeble than it should, and, at the same time, more rapid; and in all the fatal cases the pulse becomes rapid and feeble near the close of the disease; near the time of death of the patient.

The urine is, as a rule, albuminous, and diminished in amount, and the albumen may persist for sometime after the recovery of the patient from the disease.

Convalesence is often prolonged after the disease proper has run its course. After the local lesions have all disappeared the patients are often left very much prostrated; they are feeble, they are anæmic, they have no appetite, their general condition is poor, and this condition of general bad health may continue sometimes for weeks, sometimes for months after the disease proper has run its course, and it is at the commencement of convalescence that we have to look for, and to dread, death from syncope. This is a point which it is important for you to remember, that it is just at the commencement of canvalesence, when the patients are apparently likely to recover from the disease, that this danger of sudden death from failure of heart's action especially exists, and for this reason this is just the time when it is particularly important to keep these patients quiet. You should never be in too much of a hurry to let them sit up, or to let them go out after they begin to get better. The patient should be kept quiet for sometime after convalesence has fairly commenced.

There are certain regular sequelæ of diphtheria. There are, in a certain number of cases, paralysis of certain of the voluntary muscles. After the patients get well we find that there is a loss of power in certain voluntary muscles. The muscles which are paralyzed most frequently are the muscles of the soft palate, of the pharynx, and of the larynx. Next in frequency are the muscles of the eye, and here we have the involuntary muscle paralyzed, the muscle of accommodation, the ciliary muscle, and the muscle of the iris become paralyzed, and as a result of this the pupil is dilated, and the patient is unable to see near objects with any distinctness. He is able to see distinctly enough at a distance, but from the paralysis of accommodation he is unable to see anything distinctly which is close by. Besides these muscles we also less frequently have paralysis of the muscles of the arms, the muscles of the legs, the muscles of the face, and the muscles of the trunk. These paralyses are usually only temporary, they last for weeks, or they last for months, and then they gradually disappear, less frequently, however, such a paralysis may be permanent, the muscles will remain paralyzed for an indefinite period.

The prognosis of dyphtheria depends somewhat upon the particular epidemic which is prevailing, somewhat upon the age of the patient, somewhat upon whether or no the larynx is involved in the disease. There are some epidemics in which the malignant forms prevail to a very great extent, and then the larger number of the patients die. There are other epidemics in which the malignant cases are much less frequent, and then the prognosis is much better. Generally speaking there are more malignant cases during the increase of the epidemic than while the epidemic is declining. While the epidemic is increasing we usually have more malignant cases than while the epidemic is declining. The younger the child, the worse the prognosis. The younger the patients age, so much the more apt are they to die. When the larynx is involved the prognosis is always much worse, the patients are much more apt to die, and there are no cases of dyphtheria in which the prognosis is not doubtful. The disease is, at its best, a very dangerous disease. A disease which, even when you don't expect it, may cause the death of the patient.

When we come to the treatment of the disease, we have to consider the means which are adopted as local measures, and the means which are adopted as general measures, as general remedies. In the first place, in the general treatment of the disease, as to the administration of drugs, the administration of remedies which are to act on the general system. In the first place, we have alcohol. This is a favorite remedy with many. Alcohol given in the form of any of the wines, or of any of the liquors. When you speak of the alcoholic treatment of diphtheria you mean to give alcohol, not merely for the purpose of sustaining the patient, but you mean to give it so as to get the constitutional effect of alcohol upon the patient. This is what you mean by the alcoholic treatment of the disease. For this purpose you give it in large doses, and you give it in repeated doses, and you give it usually in a concentrated form ; you give it in the form of brandy, or of whiskey. To an adult you give from half an ounce to an ounce of whiskey or brandy every hour, or every half hour.

To a child you give a drachm or half a drachm of brandy or whiskey every hour, or every two hours. You give it in large quantities, in this way, with the idea of stimulating the whole system with alcohol with the idea of getting the physiological effects of alcohol upon the body. This, you observe, is a very different thing from simply giving stimulants as the patient's strength begins to diminish. This plan of giving alcohol in large and frequent doses is one which is a favorite with many practitioners, and it is one which has seemed to be successful in some places and in some epidemics.

Besides this use of alcohol in this way, we have also the ordinary use of alcohol simply as a stimulant. After the disease has lasted for some little time, and after the heart's action begins to flag, you give alcohol simply as a cardiac stimulant, as you would give it in any exhausting disease.

The use of the tincture of the chloride of iron, either by itself or alternating with doses of the chlorate of potash, is another favorite plan of treatment—a plan of treatment which has been used very extensively in this city. The ordinary way is to have made up two solutions, one solution of the muriated tincture of iron, and another solution of the chlorate of potassa. The solution of the tincture of iron is such that each dose contains from five to twenty drops, according to the age of the patient; the solution of the chlorate of potassa is such that each dose contains from thirty to sixty grains, according to the age of the patient. You give these patients alternately, every hour or every two hours; that is, one hour you give a dose of the tincture of iron, the next hour you give a dose of the chlorate of potassa.

Another plan of treatment is to give quinine, and to give it in very considerable doses. Another plan which is now not much used is to give calomel up to the point of salivation of the patient; still another plan is to give cubebs, either in the form of powder, or in the form of tincture, and to give this in considerable doses. The hyposulphate of soda in doses of from one grain to ten grains every two or three hours has also been used. Carbolic and salicylic acid in small doses have also been given internally. The benzoate of soda has been very much spoken of within the last year or so, as an internal remedy. It is given in very large doses; you give it in doses of from twenty grains to a drachm, every two or three hours; emetics are sometimes given to relieve the laryngeal dyspnœa.

These are only some of the drugs which are given, but they are the drugs which have been given most frequently, and of these the three plans of treatment which are most frequently used now, are the use of alcohol in large doses; the use of the tincture of the chloride of iron in frequently repeated doses, or the use of the benzoate of soda in large and repeated doses.

As regards the treatment, we have again a number of different substances which have been employed; and these substances may be employed locally, either by means of the brush, by means of the spray, or by means of gargles; usually we employ either the brush or the spray, for the patients can not gargle very well.

In the first place, there is the use simply of the vapor of hot water; or the vapor produced by the slacking of lime is inhaled by the patient. Of medicinal agents, the tincture of the chlorate of iron is used as a local application, either by means of the brush or by means of the spray. The nitrate of silver has been used, but is not as much used as formerly. The strong mineral acids were at one time a good deal used, but these again have fallen into disuse. The sulphate of iron in powder, alum in powder, sulphur in powder, bromine, chloral hydrate, have all been used as local applications. But the local applications, which are most frequently used, are solutions of carbolic acid and of salicylic acid. The tincture of the chlorate of iron, solutions of carbolic acid, and solutions of salicylic acid are the solutions which are most frequently applied locally, and these solutions may be applied either by means of the brush, or by means of the spray; and if they are used at all; if you expect to get any good effect from them, they should be applied very frequently; they should be applied every hour, or every two hours.

In those cases in which the larynx is involved, the question of tracheotomy always comes up. The question is always a difficult question to answer. As a matter of experience, we find that tracheotomy in these cases usually does not save the life of the patients. The patients usually die after the tracheotomy has been performed; and yet, in any given case, when you find that the patient is apparently dying simply from suffocation; simply from the laryngeal obstruction, it is very difficult; it is sometimes almost impossible to prevent yourself from performing tracheotomy, even although you know the experience is that this tracheotomy will be useless. It seems almost impossible to allow these patients to suffocate before your eyes without making at least an attempt to relieve them, so that, as a matter of fact, tracheotomy is often performed in these cases in which the larynx is involved. But it must be confessed that the results of tracheotomy are usually unsuccessfnl; the disease progresses and the patient dies.

